# Microcalcification crystallography as a potential marker of DCIS recurrence

**DOI:** 10.1038/s41598-023-33547-8

**Published:** 2023-06-08

**Authors:** Sarah B. Gosling, Emily L. Arnold, Samantha K. Davies, Hannah Cross, Ihssane Bouybayoune, Doriana Calabrese, Jayakrupakar Nallala, Sarah E. Pinder, Liping Fu, Esther H. Lips, Lorraine King, Jeffrey Marks, Allison Hall, Lars J. Grimm, Thomas Lynch, Donna Pinto, Hilary Stobart, E. Shelley Hwang, Jelle Wesseling, Kalotina Geraki, Nicholas Stone, Iain D. Lyburn, Charlene Greenwood, Keith D. Rogers, Grand Challenge PRECISION Consortium, Alastair Thompson, Serena Nik-Zainal, Elinor J. Sawyer, Helen Davies, Andrew Futreal, Nicholas Navin, Jos Jonkers, Jacco van Rheenen, Fariba Behbod, Marjanka Schmidt, Lodewyk F. A. Wessels, Daniel Rea, Proteeti Bhattacharjee, Deborah Collyar, Ellen Verschuur, Marja van Oirsouw

**Affiliations:** 1 School of Chemical and Physical Sciences, Keele University, Keele, UK; 2 Cranfield Forensic Institute, Cranfield University, Shrivenham, UK; 3 School of Cancer and Pharmaceutical Sciences, King’s College London, Guy’s Hospital, London, UK; 4 School of Physics and Astronomy, University of Exeter, Exeter, UK; 5 Division of Molecular Pathology, The Netherlands Cancer Institute, Amsterdam, The Netherlands; 6 Department of Surgery, Duke University Medical Center, Durham, NC UK; 7 Department of Pathology, University of British Colombia, Vancouver, BC Canada; 8 Department of Radiology, Duke University, Durham, NC UK; 9 DCIS411.com, San Diego, CA USA; 10 Independent Cancer Patients’ Voice, London, UK; 11 Divisions of Diagnostic Oncology, The Netherlands Cancer Institute, Amsterdam, The Netherlands; 12 Department of Pathology, Leiden University Medical Center, Leiden, The Netherlands; 13 Diamond Light Source, Harwell Science and Innovation Campus, Didcot, UK; 14 Thirlestaine Breast Centre, Gloucestershire Hospitals NHS Foundation Trust, Cheltenham, Gloucestershire UK; 15 Cobalt Medical Charity, Cheltenham, UK; 16 Baylor College of Medicine, Houston, Texas USA; 17 University of Cambridge, Cambridge, UK; 18 MD Anderson Cancer Center, Houston, USA; 19 Kansas University Medical Center, Kansas, USA; 20 University of Birmingham, Birmingham, UK; 21 Patient Advocates in Research, P.O. Box 1551, Danville, 94526-1551 California USA; 22 Borstkankervereniging Nederland, Utrecht, The Netherlands

**Keywords:** Biomarkers, Biomineralization, Breast cancer, Cancer microenvironment

## Abstract

Ductal carcinoma in-situ (DCIS) accounts for 20–25% of all new breast cancer diagnoses. DCIS has an uncertain risk of progression to invasive breast cancer and a lack of predictive biomarkers may result in relatively high levels (~ 75%) of overtreatment. To identify unique prognostic biomarkers of invasive progression, crystallographic and chemical features of DCIS microcalcifications have been explored. Samples from patients with at least 5-years of follow up and no known recurrence (174 calcifications in 67 patients) or ipsilateral invasive breast cancer recurrence (179 microcalcifications in 57 patients) were studied. Significant differences were noted between the two groups including whitlockite relative mass, hydroxyapatite and whitlockite crystal maturity and, elementally, sodium to calcium ion ratio. A preliminary predictive model for DCIS to invasive cancer progression was developed from these parameters with an AUC of 0.797. These results provide insights into the differing DCIS tissue microenvironments, and how these impact microcalcification formation.

## Introduction

Ductal carcinoma in-situ (DCIS) comprises 25% of all new breast cancer diagnoses, accounting for approximately 7000 cases per year in the UK, 50,000 in the US and 2500 in the Netherlands^1–3^. Detection of DCIS has increased rapidly since the introduction of mammographic screening due to the classic presentation of an asymptomatic area of mammographically visible calcifications. By itself, DCIS is a stage 0 form of breast cancer that has no metastatic potential. Only when DCIS progresses to invasive cancer can it lead to morbidity and mortality. However, the mechanisms of progression of DCIS to invasive breast cancer are uncertain, as the natural history of disease is poorly understood. It is estimated that invasive progression occurs in 25–35% of untreated DCIS, but to date, there are no robust biomarkers that can reliably predict progression or recurrence^4,5^. Therefore, nearly all women diagnosed with DCIS will undergo treatment in the form of surgery (± radiotherapy ± endocrine therapy) to mitigate the risk of disease progression. This inevitably leads to high levels of overtreatment^6^.

A key mammographic feature for identifying DCIS is the presence of microcalcifications, which are associated with > 85% of all DCIS cases^7^. The diagnostic and prognostic clinical advantages afforded by mammographic microcalcification are well established, with associations between some morphologies and distributions with certain disease types^8,9^. The mechanisms of microcalcification formation remain elusive although there is compelling evidence that both necrotic and active secretory (vesicle associated) pathways are implicated^10^. Regardless, evidence from in vitro studies suggest distinct mineralisation pathways associated with different microenvironments^11^.

Compositional studies have recently indicated a diagnostic advantage for disease classification by microcalcification chemistry^12^. For example, crystallographic phase appears to correlate with malignancy in breast tissue; oxalates have been uniquely associated with benign lesions while apatites are seen in both benign and malignant lesions^13,14^. More recently, studies have focussed on the structural details of apatites. When in nano-crystalline form, apatites have a remarkable ion exchange capacity and thus may provide an immortalised record of the local microenvironment physiology at the point of formation. Apatites found within breast tissues have extensive isomorphic lattice substitutions including CO_3_^2−^ substituting for both PO_4_^3−^ and OH^−^^15^. In particular, the heteroionic exchange of CO_3_^2−^ for lattice PO_4_^3−^ appears to provide a diagnostic marker for breast malignancy^16^.

During cancer development, numerous changes occur in the breast cell extracellular matrix, including the development of a hypoxic environment. Low oxygen levels in the centre of solid tumours lead to a switch to a glycolytic cell phenotype, meaning an excess of protons are produced (the Warburg effect). To maintain a neutral cell pH and prevent cell death, these protons are pumped out of the cell, causing an acidic extracellular pH. This phenomenon has been observed in many invasive cancers but also in DCIS, where the extracellular pH is found to be 6.8 compared to 7.4 in normal breast tissue^17^. A recent study has suggested that early DCIS environmental conditions can select for cells adapted to the Warburg phenotype and confer selective advantage^18^. pH has also been noted to impact the formation of calcium phosphate phases, with minerals such as whitlockite and dicalcium phosphate dihydrate being favoured over hydroxyapatite in acidic conditions^33^.

Numerous ions have also been implicated in breast cancer progression. Ca^2+^ is an important second messenger in normal cell function that can increase cell proliferation and invasion when elevated intracellularly^19,20^. Mg^2+^ is also a critical ion in breast disease development. Low Mg^2+^ levels early in carcinogenesis encourage angiogenesis and inflammation, while elevated levels at later carcinogenesis stages increase cell motility and invasiveness^21,22^. Both Ca^2+^ and Mg^2+^ are important in guiding calcium phosphate phase formation, as Mg^2+^ does not easily substitute into the hydroxyapatite lattice, leading to preferential whitlockite formation^23,24^.

Given the changes to microenvironment composition and physiology accompanying invasion, and the apparent diagnostic competence of breast microcalcification chemistry, this study aims to characterise the crystallographic attributes of microcalcifications associated with DCIS at a sub-cellular scale. Microcalcifications are examined in the tissue in situ thus retaining precise tissue-microcalcification relationships and mitigating against heterogeneous tissue inaccuracies. Further, this approach enables unequivocal phase identification and restricts interpretative models to the crystal lattice, in contrast to elemental studies where lattice and hydration layer ions are confounded. Thus, our hypothesis is that DCIS microenvironment features (e.g., cell–cell affinity, specific ion concentrations, pH etc.) that promote invasion may be immortalised within microcalcification physico-chemistry and subsequently retrieved as crystallographic biomarkers of disease progression propensity. Additionally, it may be possible to generate a new predictive model based upon the crystallographic microcalcification features that discriminate between non-progressive and progressive DCIS.

## Results

**Figure 1:**
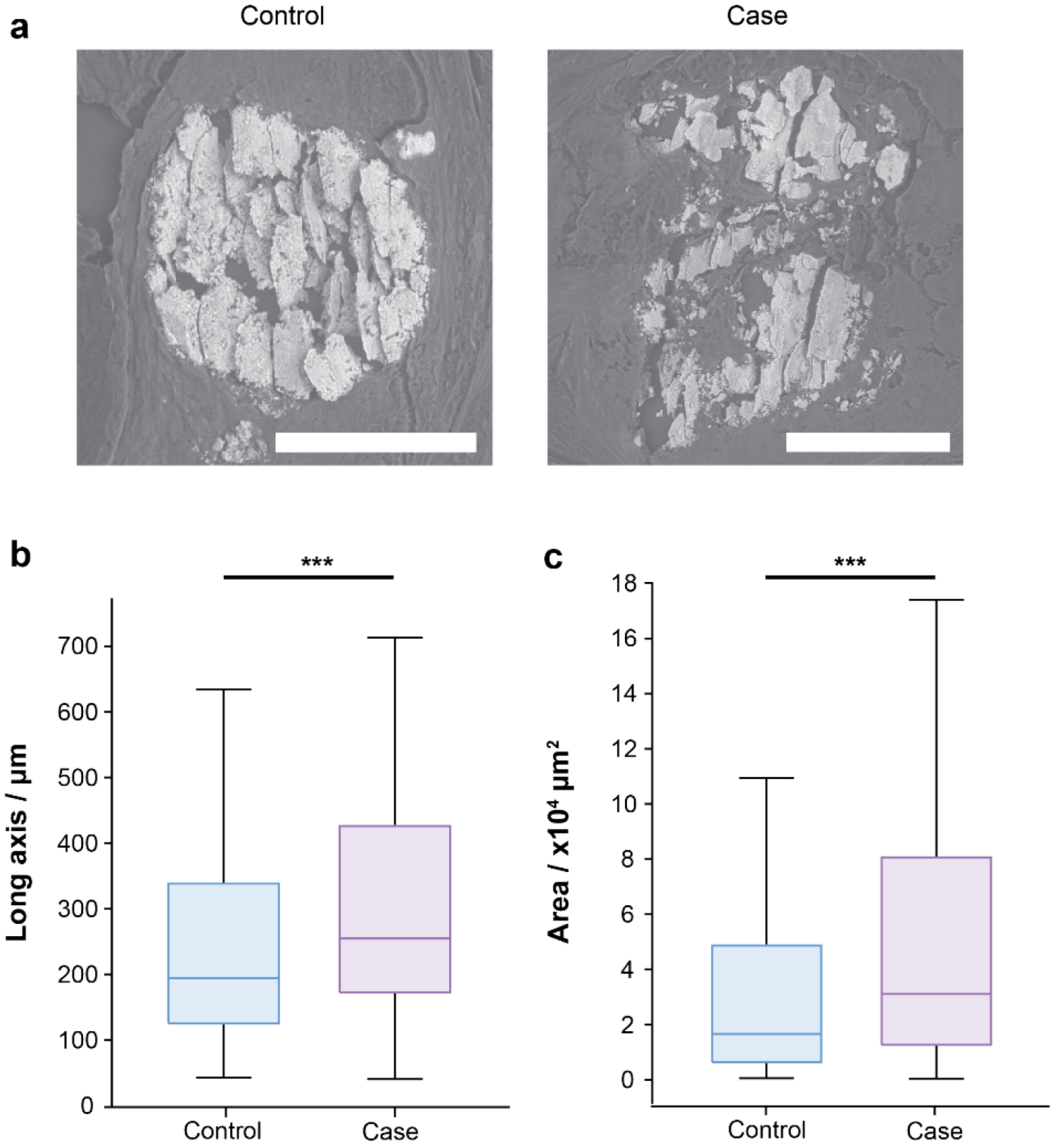
SEM images of microcalcifications. (**a**) An SEM image of an ‘average’ sized microcalcification for the control and case groups. Scale bars = 100 µm. (**b**,**c**) Box plots for the longest axis (**b**) and microcalcification area (**c**) measured from SEM images. n = 181 and 175 for control and case respectively, ***p < 0.001.

The physical dimensions of microcalcifications were measured from scanning electron micrographs (SEM). ‘Typical’ microcalcifications for control and case groups are shown in Fig. 1a. There is evidence of sectioning damage across the microcalcifications’ surfaces, but the microcalcifications remain mostly intact. Typically, microcalcifications appear as dense, white–grey deposits in SEM images, though less dense regions are present, particularly at the microcalcification edges (Fig. 1a). The longest axis was found to be significantly different (p = 0.000733) between the two groups, with case microcalcifications (246 µm, 95% CI [243 µm, 248 µm]) being, on average, ~ 33% larger compared to that of the control group (185 µm, 95% CI [183 µm, 187 µm]) (Fig. 1b). In addition, the area of the microcalcifications was also found to be significantly (p = 4.01 × 10^–4^) greater in the case group (31,112 µm^2^, 95% CI [30037 µm^2^, 32,186 µm^2^]) compared to the control group (16,629 µm^2^, 95% CI [16062 µm^2^, 17,196 µm^2^]) (Fig. 1c).

X-ray scatter experiments indicated that calcium hydroxyapatite (HAP) was the primary crystallographic phase (70–100 wt %) found in microcalcifications from both control and case groups. Additional Bragg maxima within diffractograms were consistent with the presence of the mineral whitlockite (WH), a secondary phase observed within both groups. There was no evidence of any other crystalline phases. Whole pattern fitting refinement and individual peak analysis were performed to parameterise the diffractograms and characterise the phases with respect to crystallographic features.

**Figure 2:**
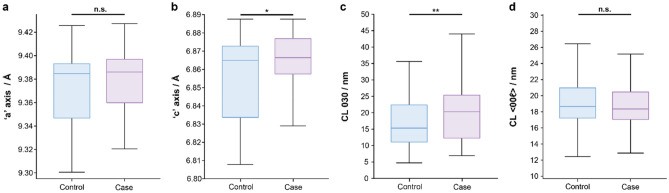
Box plots for HAP parameters determined by XRD. (**a**) ‘a’ axis lattice parameter, (**b**) ‘c’ axis lattice parameter, (**c**) CL for 030, (**d**) CL for < 00ℓ > . n = 179 and 174 for control and case respectively, *p < 0.05, **p < 0.01.

The HAP was refined in a P6_3_/m space group and both resulting ‘a’ and ‘c’ lattice parameters values were contracted compared to those for stoichiometric HAP by 0.044 Å and 0.016 Å respectively (Fig. 2a,b). Further the ‘c’ axis length was significantly reduced (p = 0.0434) in the control group (6.8649 Å, 95% CI [6.8647 Å, 6.8652 Å]) compared to the case group (6.8664 Å, 95% CI [6.8662 Å, 6.8667 Å]), while no significant difference was noted in the ‘a’ axis.

The HAP phase was also characterised by its average coherence length (CL, also domain size) that was evaluated in different crystallographic directions; < 0k0 > and < 00ℓ > , using the 030, 002 and 004 Bragg maxima respectively, with an average of 002 and 004 CL calculated for < 00ℓ > . CL was not found to significantly differ between the control and case groups in < 00ℓ > , although it was 32% greater in case group (20.30 nm, 95% CI [20.21 nm, 20.39 nm]) than the control group (15.33 nm, 95% CI [15.25 nm, 15.41 nm]) for < 0k0 > (p = 0.0012) (Fig. 2c,d). Further, CL in 202 and 310 were noted to differ significantly between the two groups (p = 0.00512 and p = 0.0459 respectively). In both cases, CL was found to be higher in the case group microcalcifications (12.20 nm, 95% CI [12.14 nm, 12.26 nm]) for 202 and 27.02 nm, 95% CI [26.93 nm, 27.11 nm] for 310) compared to the control group (11.39 nm, 95% CI [11.35 nm, 11.44 nm]) for 202 and 25.31 nm, 95% CI [25.21 nm, 25.42 nm] for 310).

**Figure 3:**
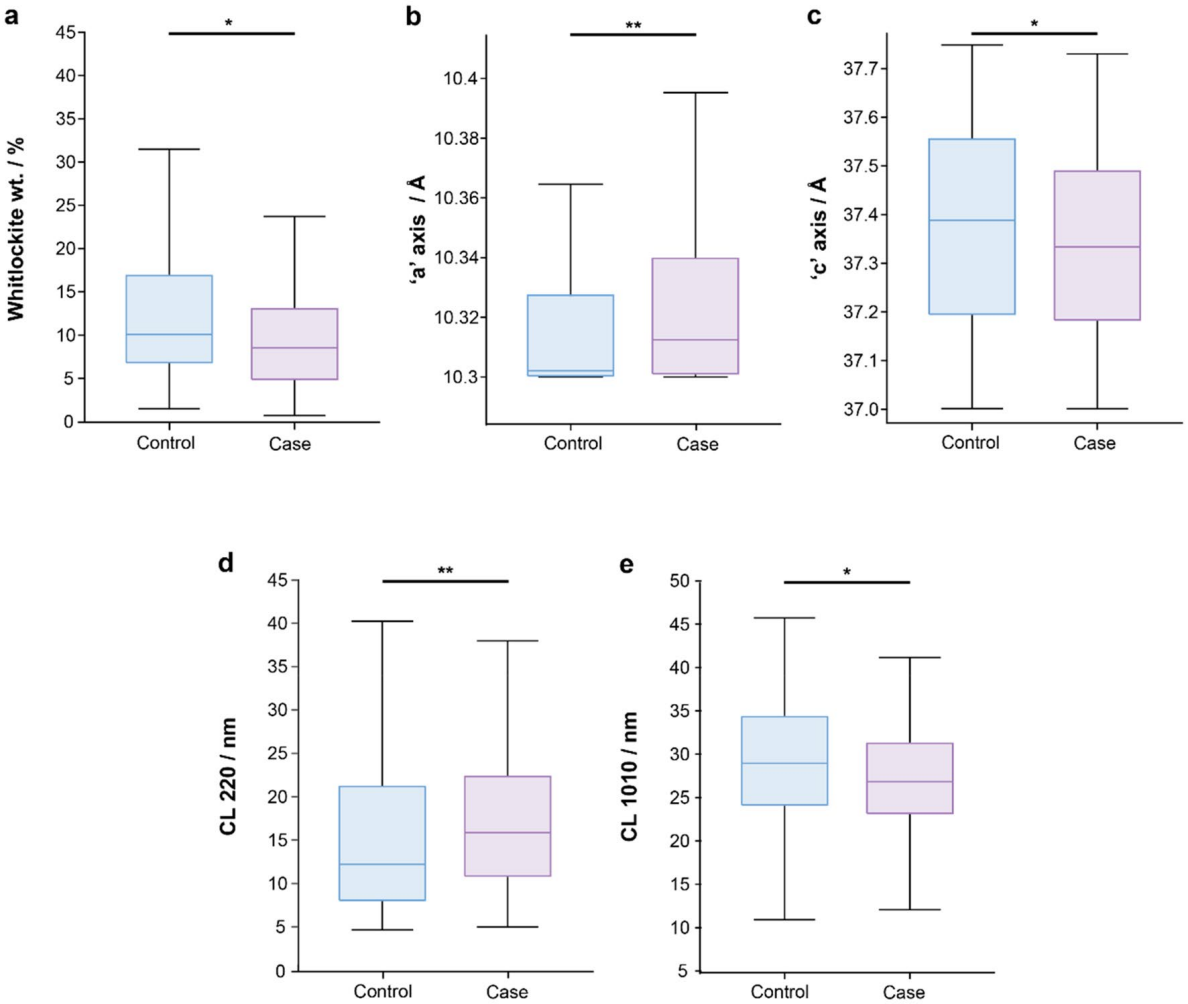
Box plots for WH parameters determined by XRD. (**a**) Whitlockite wt %, (**b**) ‘a’ axis lattice parameter, (**c**) ‘c’ axis lattice parameter, (**d**) CL for 220, (**e**) CL for 1010. n = 179 and 174 for control and case respectively, *p < 0.05, **p < 0.01.

Whole pattern analysis also enabled estimations of the relative weight percentage of WH. This was found to be significantly lower (p = 0.0237) in the case group (8.61%, 95% CI [8.53%, 8.69%]) compared to the control group (9.78%, 95% CI [9.68%, 9.87%], Fig. 3a). Crystallographic parameters of WH were also explored, with both the ‘a’ and ‘c’ axes found to be significantly different between the two groups (p = 0.00194 and 0.0127 respectively). The ‘a’ axis was found to be greater (10.3125 Å, 95% CI [10.3121 Å, 10.3129 Å]) and the ‘c’ axis foreshortened (37.3337 Å, 95% CI [37.3316 Å, 37.3358 Å]) in the case group compared to the control group (10.3046 Å, 95% CI [10.3043 Å, 10.3049 Å] and 37.3885 Å, 95% CI [37.3861 Å, 37.3909 Å] for ‘a’ and ‘c’ respectively, Fig. 3b,c).

As with HAP, the average coherence lengths were estimated for WH in different crystallographic directions. Coherence lengths for WH peaks 220 and 1010 were found to differ significantly, with CL 220 being 30% greater in the case group (15.84 nm, 95% CI [15.74 nm, 15.94 nm], p = 0.00277) versus the control group (12.22 nm, 95% CI [12.10 nm, 12.33 nm]). Conversely, CL 1010 was 7% lower in the case group (26.83 nm, 95% CI [26.75 nm, 26.91 nm], p = 0.0129) compared to the control group (28.94 nm, 95% CI [28.86 nm, 29.03 nm], Fig. 3d,e).

**Figure 4:**
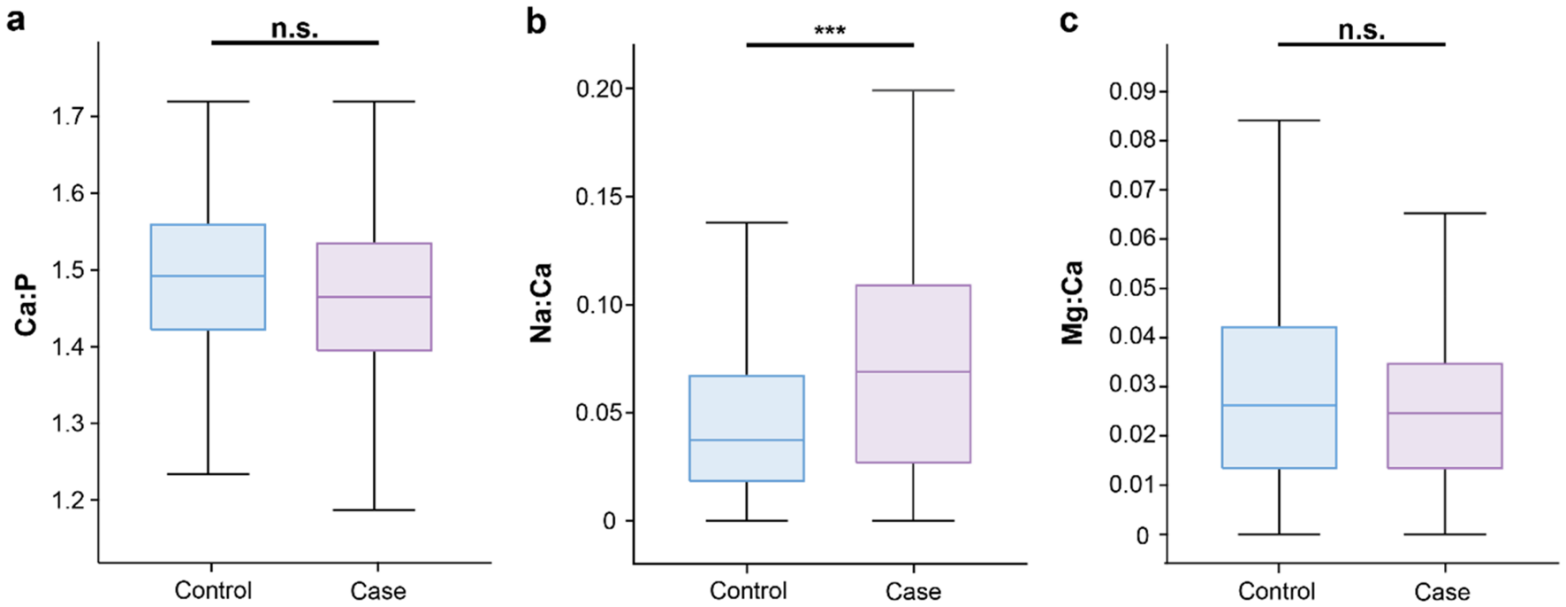
Box plots of EDX analysis of microcalcifications. (**a**) Ca:P ratio, (**b**) Na:Ca ratio, (**c**) Mg:Ca ratio. n = 179 and 174 for control and case respectively, ***p < 0.001.

Augmenting the crystallographic findings, energy dispersive X-ray spectroscopy (EDX) analysis identified calcium, phosphorous, oxygen, sodium, and magnesium within the bulk of the microcalcifications. Calibrated and quantified EDX data indicated that elemental ratios for Ca:P (1.44) were lower than for stoichiometric HAP (1.67) in both groups, but no significant difference between the control and case groups was observed (Fig. 4a). In contrast, the Na:Ca ratio was found to differ significantly (p = 0.000660) between the control (0.0374, 95% CI [0.0370, 0.0379]) and case groups (0.0689, 95% CI [0.0683, 0.0695]), with the control group having an 84% lower value (Fig. 4b). Further, Mg:Ca was not found to significantly differ between the two groups (Fig. 4c).

**Figure 5:**
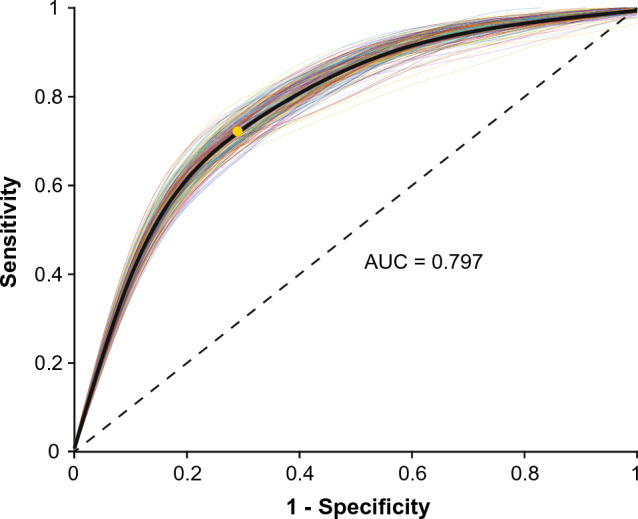
ROC curve for validation results. 200 repeats are plotted in colour, with ROC curve produced from averaging the repeats in black and a marked optimal cut point calculated from Youden’s index (yellow dot). Dotted line represents an AUC of 0.50.

From the explored parameters, eleven crystallographic and elemental features were used to build a model to predict invasive recurrence risk after DCIS, including Na:Ca ratio, lattice parameters for HAP and WH, phase relative weight percentage, CL for HAP 030, 202 and 310 and WH 1010 and 220. Linear discriminant analysis models generated ROC curves with an average AUC of 0.7972 (95% CI [0.7971, 0.7974]) (Fig. 5). Using the ROC curves produced, it is possible to achieve a sensitivity of 76% and specificity of 71% when balancing sensitivity and specificity, with an odds ratio of 7.7, a negative predictive value of 74% and positive predictive value of 73%. For a fixed high sensitivity of 95%, a specificity of 30% can be achieved, with a negative predictive value of 85% and an odds ratio of 8.9. Fixing a high specificity at 95% can yield a sensitivity of 25%, with a positive predictive value of 84% and odds ratio of 6.3.

## Discussion

The data, for the first time, indicates several significant differences in crystallographic features and chemistry of microcalcifications precipitated within DCIS lesions that do not recur and those that recur as ipsilateral invasive breast cancer. These differences support the hypothesis that different and changing microenvironments impact directly upon microcalcification growth, maturation and resorption. In general, there is evidence of significant crystallographic heterogeneity within both groups. Despite this, significant differences have been noted in both crystallographic parameters and physical dimensions of the control and case tissues. Of note, all measures of crystallite characteristics correspond to a temporal snap-shot of populations undergoing simultaneous formation and resorption/dissolution.

HAP crystallites associated with case DCIS are, on average, more mature, chemically closer to stoichiometric HAP and possess fewer lattice substitutions compared to those of control DCIS tissues. Such a population evolves from the product of preferential resorption of smaller crystallites and/or an enhanced growth enabled environment. For example, the more acidic microenvironment provided by a proliferating tumour will preferentially resorb HAP crystallites with the lowest coherence length and greatest excess lattice energy. Thus, the remaining crystallites, on average, would be expected to possess lower lattice non-uniform strain which is consistent with the greater coherence length observed for the case DCIS microcalcifications. Since a major source of non-uniform strain in biogenic apatites is carbonate, this is consistent with previous studies indicating lower carbonate within invasive tumour microcalcifications^25^. Furthermore the release of CO_3_^–2^ from the lower coherence length, resorbing crystallites will provide a local buffer environment and thus reduce the formation of phases requiring lower pH for precipitation such as whitlockite.

The greater HAP coherence lengths associated with case DCIS lesions are likely to contribute to a reduced specific surface area and therefore a diminished surface net charge on the HAP crystallites. For tumour associated microcalcification, this is consistent with increased proliferation as electrostatic cell-crystallite interactions are reduced enabling enhancement of the competitive tumour cell–cell adhesion process. This is in contrast to a previous study where the inhibition of cancer cell activity was associated with increased apatite crystallinity and morphology changes, though this study only evaluated limited maturation processes and the morphology changes (also observed within our study) are also associated with a change in apatite chemistry^26^. The morphology of HAP depends upon growth conditions and scale/type of ionic substitutions*.* Further, basal direction preferred growth has been associated with enhanced water stabilisation of the prismatic (hk0) facets. Thus the microenvironment exerts significant influence on crystallite morphology. It should also be noted that other factors have the ability to impact calcification formation and resorption, such as the use of bisphosphonates, which can prevent calcification formation, but also inhibit resorption by binding calcium phosphate crystals, when used to reduce breast cancer metastasis into the bone^27^. However, the samples used throughout this study are taken at initial DCIS diagnosis, therefore patients are unlikely to be taking bisphosphonates for this reason meaning this effect is unlikely to be present. Bisphosphonate treatment for other conditions (osteoporosis) could have a similar effect but this information was not available for the patient cohort included here.

The results indicate that a potential idiosyncratic feature of DCIS associated microcalcifications is the high prevalence of the mineral whitlockite, WH, identified in 351 of the 353 microcalcifications studied. The data demonstrates equivocal evidence of such widespread whitlockite deposition associated with DCIS, and its relationship to HAP and tissue pathology. Further, in agreement with previous studies, the WH was spatially confined to peripheral regions of the microcalcifications^28^. The formation of biogenic WH may be through direct precipitation or via conversion from HAP through multiple transient phases (these are not easily observed by X-ray scattering due to their limited mass and/or amorphous nature). HAP to WH conversion is promoted at sufficient Mg^2+^ concentrations and low pH (< 4.2). The WH/HA ratio may also be increased as Mg^2+^ is effective at blocking the hydrolysis pathway of amorphous calcium phosphate to HAP. Biogenic WH has a complex ionic structure (similar to HAP) due to multiple cation substitution at any of its 5 unique Ca^2+^ sites. Mg^2+^ is relevant in the context of tumour progression as it is unlikely to be substituted within the HAP lattice due to its decreased ionic radius (0.069 nm) compared to calcium (0.099 nm), which would destabilise the HAP lattice^23,24^. Substitution of Ca^2+^ by Mg^2+^ within WH causes a Vegard lattice compression but other ionic exchanges (e.g. Na^+^, Fe^2+^) simultaneously cause expansion.

Magnesium also plays multiple roles in cellular functions including DNA repair, antioxidant functions and genomic stability^21^. At early stages of breast cancer development, intracellular magnesium levels are depleted, leading to an increase in the inflammatory response which in turn triggers angiogenesis^29^. Magnesium depletion can also lead to a loss of DNA repair mechanisms, leading to increased genetic mutations. Later in tumour development, magnesium is required to facilitate processes involved in cell proliferation and migration, therefore the intracellular magnesium levels are increased through the overexpression of TRPM7 and CNNM3 magnesium ion channels^21^.

We surmise that the sequestering of Mg^2+^ and its concomitant depletion from the microenvironment by WH will influence the cellular dynamics at multiple stages of tumour development. For both control and case DCIS lesions, the formation of WH at the microcalcification periphery impedes further growth of HAP nanocrystallites but this occurs at a later stage of microcalcification enlargement and HAP maturity for the case group. Therefore, while a greater or similar WH mass may be associated with the case DCIS microcalcifications compared to the control group, averaged across the larger microcalcification area this appears to be a lower relative weight%.

The values of Na:Ca, as measured by EDX and averaged for each microcalcification, are consistent with those reported previously^30^. The greater value for the case group microcalcifications may appear inconsistent with the model presented above in that it suggests greater carbonate within this group; Na^+^ for Ca^2+^ charge balance coupling to accommodate CO_3_^2−^ for PO_4_^3−^ exchange. However, the elemental measurements were averaged for each microcalcification and thus provide compound elemental ratios for both HAP and WH. The WH lattice also accommodates Na^+^ in heteroionic exchange for Ca^2+^ and the result is an increase in ‘a’ and decrease in ‘c’ which is consistent with our observations. Thus, it is likely that this WH contains significant amount of sodium.

This work has provided interesting insights into the potential microcalcification formation mechanisms occurring in DCIS lesions, but perhaps most importantly, allowed the development of a potential model to predict DCIS invasive recurrence risk using microcalcification phase crystallography. AUCs from the ROC curves produced in this work are similar to radiologist’s performance analysis to predict DCIS upstaging (AUC = 0.765)^31^. Thus, this work complements current predictive methods that could improve DCIS recurrence predictions by identifying additional biomarkers. While these parameters are not directly measurable using current histopathological laboratory equipment, future technological advances in X-ray systems, may permit the direct interrogation of these features as part of a standard pathological lab. Using the ROC curves produced, it is possible to achieve a sensitivity of 76% and specificity of 71%, which, if employed across the UK, USA and Netherlands, using the case numbers previously stated, could correctly classify ~ 43,000 DCIS cases as having the potential to recur, or being likely to not recur at the time of initial biopsy. This work could identify women in need of further intervention to prevent these recurrences and sets the foundation for further work in identifying calcification biomarkers in progressive DCIS, as well as recurrent.

## Methods

### Samples

For XRD and EDX analysis, 353 microcalcifications from the formalin fixed paraffin embedded (FFPE) breast tissue sample blocks in 124 patients were selected. Cases of primary calcified DCIS from Duke University (4 patients), The Sloane Project, collated by Kings College, London (49 patients) and the Netherlands Cancer Institute (71 patients) were selected. Ethical approval was received for all samples from NHS Health Research Authority, REC number 18/LO/0945. In all cases, consent was not required for the samples.

For samples obtained from Duke University, consent was waived as part of the Duke University Health System Institutional Review Board, numbers: Pro00054515 and Pro00054877, in compliance with HIPAA regulations. For samples from the Sloane Project (Ethical approval REF 08/S0703/147, 19/LO/0648), ethics approval and consent to participate Ethics Committee approval was not required, originally conducted under the NHS Cancer Screening Programme’s application to the Patient Information Advisory Group (PIAG). For samples from NKI, the study was approved by the review boards of the Netherlands Cancer Registry and the Dutch National Pathology Automated Archive (PALGA). The secondary use of tissue and data in this study is covered by an opt-out regimen conform Dutch regulations, the Code of Conduct of Federa-COREON (Federa-COREON. Dutch Regulations and the Code of Conduct (Federa-COREON, 2004)) and the international Guideline on Good Clinical Practice. The study also meets the General Data Protection Regulation (GDPR) criteria that came into effect on 25 May 2018.

All samples were taken at initial DCIS diagnosis (not at recurrence), where patients received subsequent treatment. Each patient had at least 5 years of follow up with either no known recurrence (174 microcalcifications in 67 patients) or ipsilateral invasive breast cancer recurrence (179 microcalcifications in 57 patients), herein referred to as control and case. Patient ages ranged from 36 to 81 years old, with median age in the control group of 60 (36–81 y.o.) and in the case group of 57 (38–76 y.o.). No significant difference was noted between the two groups (p = 0.325, Kruskal–Wallis test), therefore age was not further considered as a confounding variable in this work.

Between 1 and 3 microcalcifications were randomly selected from each sample using the inclusion criteria that: microcalcifications were located within the region of DCIS, identified through haematoxylin and eosin-stained slides; and the selected microcalcifications were present on the sections cut for X-ray diffraction (XRD), confirmed through SEM.

Samples were distributed in similar ratios across DCIS grades 1, 2 and 3 in both the control and case groups, as shown in Table 1 below. No significant differences were observed between the grade distributions in the two groups (p = 0.7700, Kruskal–Wallis test).

**Table 1:** Number of calcifications (and samples in brackets) per DCIS grade in the control and case groups.

	Control group	Case group	Total
DCIS grade 1	13 (6)	18 (6)	31 (12)
DCIS grade 2	70 (27)	66 (21)	136 (48)
DCIS grade 3	91 (34)	95 (30)	186 (64)
Total	174 (67)	179 (57)	353 (124)

5 μm thick FFPE sections were mounted onto 12.5 μm thick polyolefin substrate stretched over 38 mm diameter aluminium rings and held in place with rubber rings. The same samples were used for both XRD and EDX.

Microcalcification size measurements (long axis and area) were taken for three hundred and fifty-six microcalcifications across 135 tissue samples: 181 microcalcifications in 56 control samples and 175 microcalcifications in 51 invasive case samples. These numbers differ to the sample sizes for XRD and EDX analysis, due to extensive calcification loss between SEM imaging and subsequent analyses in a small number of cases. Calcification cut using standard histological processing is very fragile, due to the differences in tissue hardness.

### X-ray diffraction

**Figure 6:**
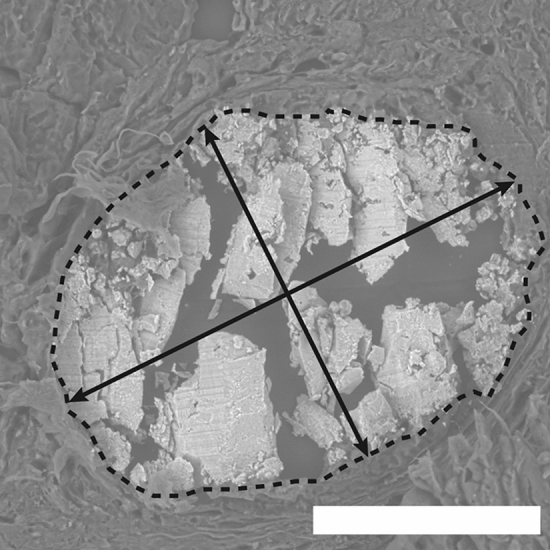
Measurements taken across microcalcifications. XRD data was collected along the longest axis and a perpendicular axis passing through the centre, at 11 µm intervals, marked as double headed black arrows. The longest axis was also measured from SEM images for size analysis. Microcalcification area was measured by outlining each microcalcification (dotted line). The same area was used for EDX analysis. Scale bar = 100 µm.

XRD data was collected on the i18 beamline at Diamond Light Source, Didcot UK, using a beam energy of 12 keV, spot size of 5 × 5 μm and an Excalibur detector. Samples were mounted normal to the X-ray beam. Regions of interest were identified using a microscope mounted at 45° to the sample stage, and the presence of calcium was confirmed through X-ray fluorescence experiments at 5 keV using a Vortex Silicon Drift Detector. Two orthogonal lines were collected across each microcalcification, using a step size of 11 μm and collection time of 15 s per point. The first line was plotted across the longest axis at the centre of the microcalcification, and the second was plotted perpendicular to the first, again across the microcalcification centre (Fig. 6).

2-D data was azimuthally integrated into 1-D data using the Diamond Analysis WorkbeNch (DAWN) software (V2.18.0, Diamond Light Source)^32^. Phase identification was carried out using the International Center for Diffraction Data (ICDD) database (PDF-4, 2018) and microstructural analysis was carried out using Topas Academic (V6.1, BRUXER AXS).

Whole pattern analysis of data permitted the fitting of space groups P6_3_/m and R3c to the HAP and WH phases respectively and refinement of the unit cell contents. Such refinement involves a least squares approach to fit a predefined pattern to the presented data, allowing the refinement of relative weights of each phase, as well as lattice parameters.

Coherence length, CL, (or domain size) represents the average distance within a crystal over which lattice order persists and is often employed as a measure of ‘crystallinity’ for XRD data. CL can be quantified using the Scherrer equation:

$$CL=\frac{K\lambda }{{\beta }_{hkl}cos{\theta }_{hkl}},$$

where K is the shape factor (0.9, the value commonly used for this constant), λ is the wavelength (0.1033 nm), β_hkl_ is the FWHM (assuming no instrumental broadening from the synchrotron instrumentation) and θ is the Bragg angle.

CL is distinct from ‘crystallite size’, as it is composed of both crystallite size and non-uniform strain components, as outlined by Williamson and Hall^33^.

### Energy dispersive X-ray spectroscopy

SEM and EDX were carried out by attaching FFPE samples prepared as described above, onto a 51 mm aluminium stub with copper tape to increase the surface conductivity. Images and elemental data were collected using a Hitachi SU3500 system with an 11 keV beam energy and 70 Pa vacuum in variable pressure (VP-SEM) mode. For EDX, a working distance of 10 mm was used, and average measurements were collected across each microcalcification for 60 s (Fig. 6). Data was analysed using EDAX TEAM software (V4.5) to determine elements present and calculate elemental ratios.

SEM images were analysed using ImageJ (V1.53c, National Institutes of Health)^34^, to measure the microcalcification long axis and microcalcification area (Fig. 6) and calculate the roundness and circularity shape descriptors of the microcalcifications.

### Data processing and statistical analysis

For XRD, where multiple measurements per microcalcification were collected, a median value for each parameter was calculated before matching data to the equivalent EDX and SEM measurements. Median values were calculated for each recurrence class (no recurrence and ipsilateral invasive recurrence), and box plots produced in Matlab (Mathworks, R2021a).

Each parameter was tested for normality using a Shapiro–Wilk test, with all parameters found to not be normal in distribution. Subsequent Kruskal–Wallis tests were performed to determine significant differences (95%) between the two groups, with all p values and 95% confidence intervals (CI) reported as a result of these tests.

### Data modelling

Median values for significantly different XRD and EDX parameters for each microcalcification (n = 353) were used to build a training model, including HAP and WH lattice parameters, WH weight percentage, CL for HAP 030, 202 and 310 and WH 1010 and 220. A predictive model was built using linear discriminant analysis in Matlab (Mathworks, R2021a). Inputted data was normalised ahead of data modelling to satisfy the normality assumptions of linear discriminant analysis. Models were validated using fivefold cross validation of the training data, randomly repeated 200 times and an average model calculated. Cross validation sets were separated by patient, ensuring that microcalcifications from the same patient were not included in both the training and validation sets.

## Data Availability

Data is available on request from the corresponding authors (SG and KR).
